# Clinical Outcome and Cost-Effectiveness Analysis of CSII Versus MDI in Children and Adolescent With Type 1 Diabetes Mellitus in a Public Health Care System of China

**DOI:** 10.3389/fendo.2021.604028

**Published:** 2021-03-30

**Authors:** Sicui Hu, Hongxiu Yang, Zhihong Chen, Xuefei Leng, Cheng Li, Lingyan Qiao, Weiqing Lv, Tang Li

**Affiliations:** ^1^ Paediatric Endocrinology and Metabolism Department, Qingdao Women and Children’s Hospital, Qingdao, China; ^2^ Neuroendocrine Pediatric Department, The Affiliated Hospital of Qingdao University, Qingdao, China; ^3^ Research and Development Department, Medtronic (Shanghai) Management Co, Ltd, Shanghai, China

**Keywords:** type 1 diabetes mellitus, continuous subcutaneous insulin infusion, multiple daily injections, glycated hemoglobin, cost-effectiveness analysis, quality adjusted life years, incremental costs and effectiveness ratio

## Abstract

**Objectives:**

To evaluate the clinical and economic consequences of continuous subcutaneous insulin infusion (CSII) vs. multiple daily injections (MDI) in children and adolescents with type 1 diabetes mellitus (T1DM) from a public health care system in developed areas of developing country, considering changes in glycemic Control, daily insulin requirements, lipid profile, body mass index (BMI), frequency of severe hypoglycemia and Diabetic Ketoacidosis (DKA) and diabetic complications.

**Methods:**

This was a retrospective cohort study of children and adolescents with T1DM. Data were collected at baseline and the end of every year including glycated hemoglobin (HbA1c), insulin dose, lipid profile, blood pressure, and adverse events (severe hypoglycemia and DKA). The Cost-effectiveness analysis was performed using the IQVIA CORE Diabetes Model (CDM) to simulate diabetes progression by utilizing the clinical data obtained from the two groups. The main outcome measures were Life Expectancy, Quality adjusted life years (QALYs), Total Costs and Incremental Costs and Effectiveness Ratio (ICER) of CSII compared with MDI in Chinese pediatric patients with T1DM in Qingdao City (60 years).

**Results:**

Mean HbA1c values and daily insulin doses were significantly lower in those receiving CSII therapy throughout follow-up. Mean direct lifetime costs were ¥ 67,137 higher with CSII treatment than with MDI for pediatric patients. Treatment with CSII was associated with an improvement in life expectancy of 0.41 years for pediatric patients compared with MDI based on CORE diabetes model simulation. The corresponding gains in QALYs were 0.42. These data produced corresponding ICER is ¥ 161,815 per QALY for pediatric T1DM patients in Qingdao. Sensitivity analyses suggested that our base-case assumptions were mostly robust.

**Conclusions:**

CSII is associated with improved long‐term clinical outcomes compared with MDI. Based on this model analysis, CSII appears to be more cost-effective for the Qingdao TIDM pediatric population and health care system.

## Introduction

T1DM accounts for 90% of all types of childhood diabetes. It is a major pediatric endocrine disorder with a prevalence rate of 2/100,000~5/100,000 in recent years in China. The incidence rate of <5 years old children increased by an average of 5%~34% a year, suggesting that an decreasing age at onset of the disease (1, 2). The compliance rate of blood glucose control in children with T1DM in China is far lower than that in western countries. The epidemiological data of long-term blood glucose control and prognosis of childhood T1DM in China are scarce. The only multicenter study in 2006 showed that the average HbA1c was 9.5%, and the HbA1c standard rate of single center in China was only 15%, far lower than 44%~59% of European countries such as the United States and Germany (3, 4).

In the 1993 research results of Diabetes Control and Complications Trial (DCCT) show that intensive insulin therapy can reduce hyperglycemia, improve the quality of life, prevent the development and progression of future microvascular and macrovascular complications (5). There are two possible options for intensive insulin therapy: MDI and CSII. Intensive insulin treatment in the form of MDI has been the standard treatment method in most clinical practice institutions worldwide (6). Especially in developing countries, MDI was often used in TIDM for initial therapeutic maneuvers in both children and adolescents patients. However, there are some difficulties occur in the most optimal glycemic control since compliance with MDI therapy and meal compliance is lower during child and adolescence than in adulthood, so only a small percentage of patients achieve desired glycemic targets with MDI therapy (7). CSII is an alternative intensive treatment to MDI, representing the most physiological insulin therapy currently available in China, more closely simulating physiological meal-independent insulin secretion and is also reported to achieve a significantly lower risk of hypoglycemic attack with no increase the risk of diabetic ketoacidosis (8). Furthermore, the effectiveness of CSII therapy has been confirmed by randomized controlled studies and meta-analyses of various observations and in childhood and adolescence studies (9).

CSII has only been used in pediatric patients since 2000, although CSII therapy has been available in the treatment of T1DM since the 1970’s (10). Compared with MDI, CSII is significantly more expensive and has not been widely used in China. So there are still significant knowledge gaps regarding clinical and economic outcomes in China. Recently, there has been a significant increase in CSII use in children and adolescents in Qingdao, China, because the reimbursement policy through special medical device aid project for serious and rare diseases for all Qingdao children and adolescents with T1DM. 80% reimbursement for Medtronic paradigm 722 pump system for pediatric T1DM (<19 years old) in Qingdao area, and infusion sets are covered as well.

The purpose of this study is to assess the effect of long term CSII treatment for pediatric T1DM in Qingdao, China and to compare both the efficacy, assessed by HbA1c levels, insulin dosage and BMI, and safety, assessed by ketoacidosis and hypoglycemia incidence. Finally, the cost-effectiveness analysis of CSII vs. MDI therapy will be presented.

## Patients and Methods

### Study Design

This was a retrospective, controlled observational study comparing Glycemic Control, Insulin Dose, Plasma Lipid Metabolism and BMI between the MDI group and the CSII group. The clinical data obtained from these pediatric T1DM patients were used for cost-effectiveness analysis. Through CDM calculate the economic data such as QALY, ICER, and long-term treatment costs, and evaluate the health economic benefits of the Qingdao project. Considering that T1DM is a life-long progressive disease, in order to simulate the lifetime treatment of patients, the research time limit was set as 60 years. The model cycle was 1 year and the future treatment cost and clinical effect were discounted at a discount rate of 5% per year.

### Construction of Health Economics Model

The model consists of several interrelated Markov sub-models. These sub-models divide the disease into different states and simulate the progression of diabetes and its complications (including angina pectoris, myocardial infarction, congestive heart, stroke, peripheral angiopathy, diabetic retinopathy, macular edema, cataract, hypoglycemia, ketoacidosis, nephropathy (including microalbuminuria, massive albuminuria and end-stage renal disease), neuropathy, foot ulcer and amputation, pulmonary edema, depression, and non-specific mortality) through Monte Carlo method. Finally, the data were entered into the CORE diabetes model for simulation (60 years), and the expected life, quality-adjusted life years and total cost were obtained under different intervention conditions, so as to carry out health economic evaluation.

### Population

Between January 2015 and May 2019, 156 patients aged between 2 and 18 years, with T1DM underwent insulin pump initiation in Qingdao area. Data on 104 subjects were retrospectively included in the study analysis. Each patient in the CSII group was matched for age, sex, diabetes duration, insulin requirement and metabolism control with a patient on MDI (n=104). T1DM was diagnosed based on International Society of Pediatric and Adolescent Diabetes/International Diabetes Federation (ISPAD/IDF) guidelines.

Qing Dao Women and Children’s Hospital Ethical Committee Approval Was Obtained for the Study (QFELL-KY-2019-61).

### Population Management

Paradigm 722 and Veo Medtronic MiniMed Solution pump with CareLink™ (Medtronic MiniMed, Inc) were used in the CSII group. All pumps except six low weight patients (used general insulin) used ultra-short acting insulin, aspart. CSII data for all cases were transferred from pump data to computer (CareLink^®^ Pro Therapy Management Software, MiniMed Medtronic) at each visit. The MDI protocol (four injections per day) consisted of long and ultra-short acting (or Regular and NPH insulin) analogs (detemir and aspart).

Patients were seen routinely every two to 3 months during MDI and after the pump therapy stabilization period. BMI, insulin dose, HbA1c, lipid profile and blood pressure were evaluated at baseline and every 3 months for up to 4 years. Insulin dose were calculated at each visit for all patients. HbA1c and lipid profile were measured by using the Siemens ADIVA 2400 Analyzer (Siemens Healthineers, Inc). Severe hypoglycemic episodes were defined as seizures and/or reduced levels of consciousness needing for assistance from others and hospitalization. The incidence of severe hypoglycemia episodes was calculated as the number of episodes per 100 patients per year.

### Data Sources

Demographic (gender, height, weight, age at diagnosis, age at intervention, diabetes duration, and socioeconomic status) and clinical (HbA1c, insulin doses, BMI, lipid profile and blood pressure) data were derived from electronic medical records. Frequency of capillary blood glucose monitoring, incidence of hypoglycemic attacks and episodes of diabetic ketoacidosis were obtained retrospectively from file data recorded at every 3-monthly clinic visit. Cost data was obtained by searching online pharmacies and literature review (11, 12) and calculation of treatment cost based on local product cost.

### Statistical Analysis

All statistical analyses were carried out using IBM SPSS Statistics version 25. P-value less than 0.05 was considered to be statistically significant. Data were expressed as mean and standard deviation (SD) with 95% confidence intervals (CI).The normal distribution of the data was tested by Kolmogorov-Smirnov test. Chi-square analysis was used to analyze the classified variables. Student’s t-test was used to compare the mean of two normally distributed variables. The median of continuous variables (quartile range) was used to calculate the descriptive statistics of demographic and clinical factors, and number with percent for categorical variables. Pearson correlation analysis was used to evaluate the linear correlation between variables.

## Results

### Demographic Data

Baseline characteristics of CSII and MDI groups are shown in **Table 1**. There was no statistical difference in terms of mean age of the subjects, gender distribution, insulin dose, biochemistry indicators and diabetes duration between the two groups.

**Table 1:** Baseline characteristics of the study population using CSII and MDI.

Characteristic	CSII group (104)		MDI group (104)		P value
	Mean	Standard deviation	Mean	Standard deviation	
**Demographic information**					
Age(years)	10.32	3.84	9.86	3.91	0.173
Diabetes duration (years)	3.64	2.41	3.11	1.3	0.143
CSII treatment time (years)	2.63	1.14	NA	NA	NA
Insulin dose(U/kg/d)	0.98	0.17	0.96	0.14	0.333
Male ratio	48.62%		44.23%		0.521
**Baseline risk factors**					
HbA1c (%)	12.68	3.03	12.97	2.81	0.594
Systolic blood pressure (mmHg)	107.58	12.57	104.55	11.68	0.099
Diastolic blood pressure (mmHg)	68.02	9.62	66.86	10.04	0.437
Total cholesterol (mg/dl)	180.78	50.74	179.95	58.62	0.923
High density lipoprotein (mg/dl)	48.86	11.93	48.70	15.12	0.941
Low density lipoprotein (mg/dl)	108.15	38.19	100.24	36.61	0.183
Triglyceride (mg/dl)	110.80	107.68	130.09	135.77	0.320
BMI (kg/m^2^)	17.98	3.01	18.03	2.97	0.718
Hemoglobin (g/L)	132.26	13.34	130.84	18.58	0.565
White blood cells (10^6/^ml)	8.05	4.64	10.53	12.36	0.063
Heart rate (heartbeats per minute)	97.47	17.84	102.25	18.15	0.079
Creatinine serum (mg/dl)	57.20	19.61	49.37	23.83	0.018
Albumin serum (g/dl)	39.18	5.60	39.16	5.49	0.980

Data expressed as mean ± SD.

### Clinical Effective Data of CSII and MDI Groups

#### Metabolic Control

**Figure 1:**
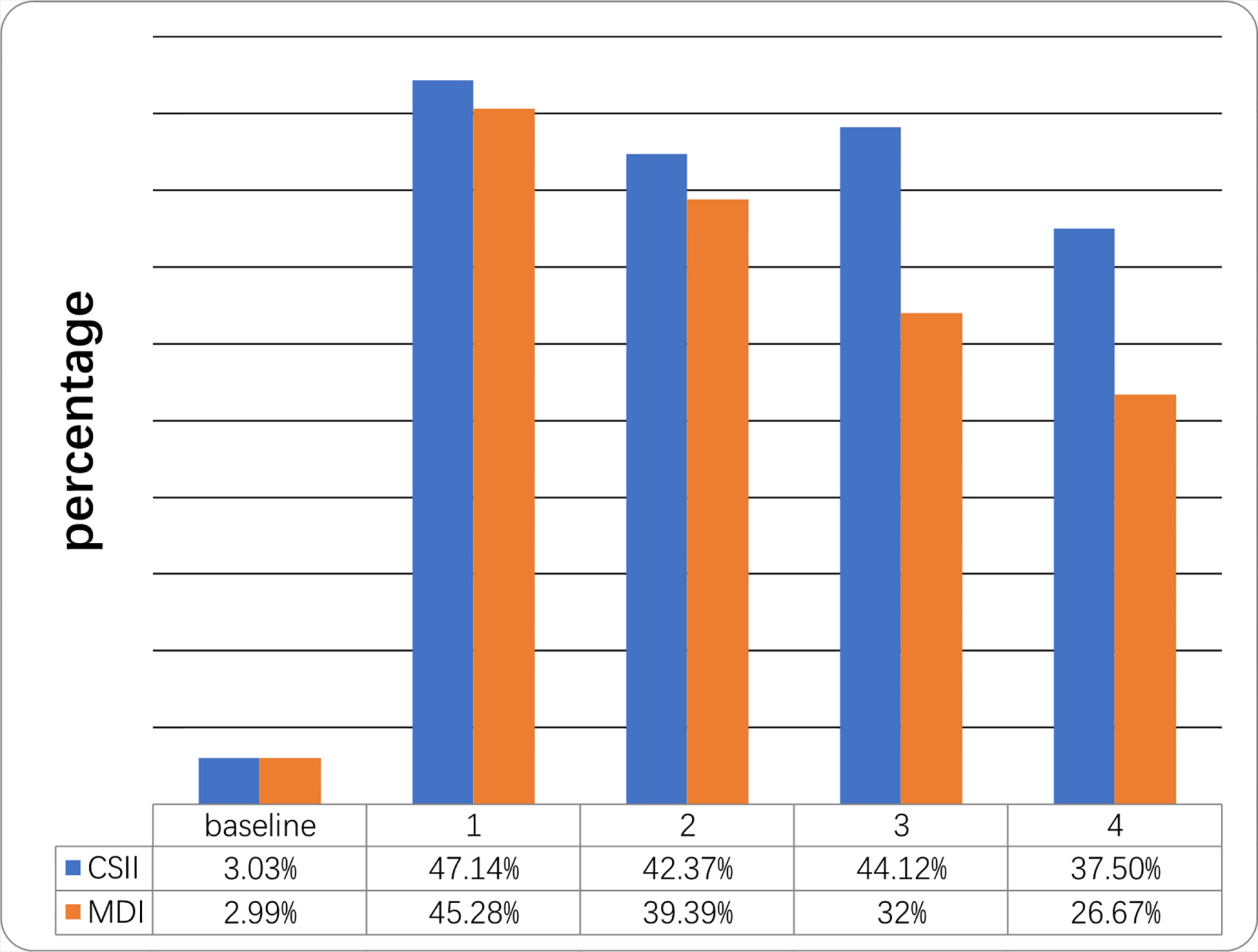
Percentage of children who reached the target of HbA1c <7.5% at different time points during the 4-year follow-up. Follow-up (years).

Mean HbA1c in the year prior to initiation of CSII was 12.68 ± 3.03% while mean HbA1c at the end of the first year was 7.79 ± 1.87%. HbA1c levels were <7.5% in 3.3% and 47.14% of cases respectively. Mean HbA1c at the end of the 4th year was 8.85 ± 3.96% and 12 patients (37.5%) still had a mean HbA1c <7.5% in the CSII group. Mean initial and 4-year HbA1c levels on MDI therapy were 12.97 ± 2.81% and 10.47 ± 0.36% respectively and four (26.67%) of the patients’ HbA1c were <7.5% at the end of 4th year (**Table 2** and **Figure 1**). The compliance rates of HbA1c in the CSII group was higher than MDI throughout the study period. Mean HbA1c was significantly lower in the CSII group during the 4 years follow-up. No correlation was found between HbA1c levels and sex, duration of CSII or insulin doses used.

**Table 2:** HbA1c difference during the 4 years follow up in continuous subcutaneous insulin infusion and multiple daily insulin groups.

	Baseline	1st year	2nd year	3rd year	4th year
CSII group					
Mean HbA1c	12.68 ± 3.03%	7.79 ± 1.87%	8.1 ± 0.7%	8.68 ± 2.02%	8.85 ± 3.96%
N(104)	91	70	59	34	32
MDI group					
Mean HbA1c	12.97 ± 2.81%	8.52 ± 2.03%	8.97 ± 2.4%	10.23 ± 3.24%	10.47 ± 0.36%
N(104)	75	53	33	25	15
HbA1c difference (CSII-MDI)	-0.29	-0.73	-0.87	-1.55	-1.62
P value	0.498	0.043	0.052	0.105	0.169

#### Insulin Dosage

**Figure 2:**
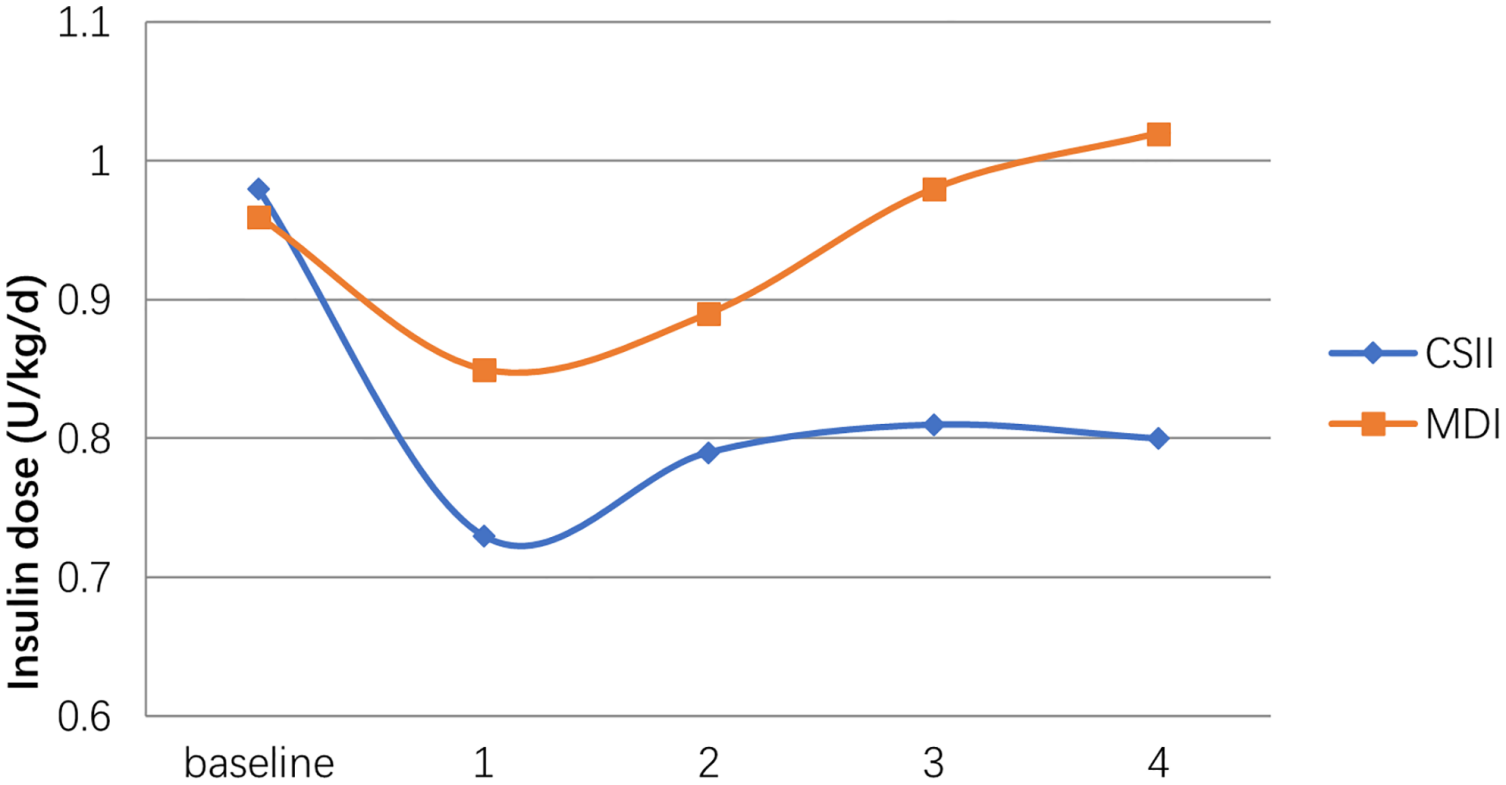
Daily insulin requirement changes in both CSII and MDI groups.

Children using MDI therapy used lower total daily insulin doses compared to those treated with CSII at the beginning of therapy (MDI: 0.96 ± 0.14 U/kg/d; CSII: 0.98 ± 0.17 U/kg/d respectively). Insulin dose in the first year of treatment was 0.73 ± 0.20 U/kg/d in the CSII group and 0.85 ± 0.16 U/kg/d in the MDI group. At the end of the 4th year, the insulin requirement of MDI group was higher than that of baseline. In contrast, the dose of CSII group at the same time point was significantly lower than the baseline. Insulin dose was significantly lower in the CSII group compared to the MDI group in all of the time periods (p<0.05; **Figure 2**).

#### Lipid Profile

When the mean lipid profiles (triglyceride, total cholesterol, HDL- and LDL-cholesterol) of the patients following CSII group were compared with MDI group, there was no statistically significant difference (p >0.05; **Table 3**). Only three patients (two aged 15 and one aged 17 years old) had mild elevation in their total cholesterol levels. Their triglycerides were normal. Two were in the CSII group and one in the MDI group. Due to the short follow-up period, no obvious complications were observed in the two groups, and there were no significant changes in blood pressure, glomerular filtration rate and other risk factors.

**Table 3:** Plasma lipid parameters of the two groups in the 1st and 4th years.

	CSII group	MDI group	P
**End of the 1st year**			
Triglycerides (mg/dl)	80.55 ± 39.10	74.92 ± 34.85	0.723
Total cholesterol (mg/dl)	173.38 ± 39.67	164.78 ± 42.75	0.477
HDL-cholesterol (mg/dl)	47.18 ± 11.84	49.27 ± 14.96	0.493
LDL-cholesterol (mg/dl)	103.41 ± 30.03	95.88 ± 35.55	0.151
**End of the 4th year**			
Triglyccridc (mg/dl)	68.07 ± 32.22	73.86 ± 36.98	0.968
Total cholesterol (mg/dl)	149.42 ± 26.54	144.91 ± 26.21	0.333
HDL-cholesterol (mg/dl)	69.39 ± 14.59	68.86 ± 13.58	0.634
LDL-cholesterol (mg/dl)	79.43 ± 15.62	71.63 ± 18.26	0.6

#### Adverse Events

Diabetic ketoacidosis was observed in one of the CSII cases (0.89/100-patient-years) and three of the patients on MDI treatment (2.46/100-patient-years) during monitoring. Severe hypoglycemia decreased from 9.6 events/100-patient-years at baseline to 1.5 events/100-patient-years at the end of the follow up period in the CSII group (p=0.036). However, it increased from 9.1 events/100-patient-years at baseline to 18.7 events/100-patient-years at the end of the study in the MDI group (p=0.042).

### Health Economic Data

#### Treatment Cost Calculation

The price of insulin pump is about 60,000 RMB. Supposed the upgrade cost remain unchanged for life time. Based on the average life of pump upgrading is 7.7 years, if patients use insulin pump for 60 years, the average annual cost of the pump is ¥7792.2.Calculation process: Annual cost of the pump =[60,000 + 60,000*(60-7.7)/7.7]/60=¥7792.2 (RMB). **Table 4** shows the treatment cost of CSII and MDI groups.

**Table 4:** The treatment cost of CSII and MDI groups.

Item	Cost (RMB)
CSII Group	
Initial price of insulin pump^1^	¥60,000
Upgrade costs^1^	¥60,000
Calculated Average annual cost^1^	¥7792.2
Average annual cost of pipeline consumables^1^	¥7741
Insulin Aspart per year^2^	¥2,465
CSII Average annual total expenses of the group	¥17,998
MDI Group	
Average annual cost of insulin glargine (basic)^3^	¥5,027
Insulin Aspart per year^2^	¥1,541
Average annual cost of insulin pen^4^	¥19.80
Average annual cost of needles^4^	¥749.60
MDI Average annual total expenses of the group	¥7,337

Exchange Rate$1=¥6.95, 1) Provided by the manufacture; 2) Yaozhi Network: https://db.yaozh.com/; 3) Jing Wu, Xiaoning He, Yanhui Liu. Cost-Effectiveness Analysis of Insulin Aspart 30 Versus Insulin Glargine in Patients with Type 2 Diabetes in China. Chinese Pharmaceutical journal,2016;51(03):242-247; 4) Online pharmacy. Jingdong Pharmacy: https://mall.jd.com/index-1000015441.Htm.

#### Base Analysis Results

The total direct medical costs of patients in CSII group and MDI group for 60 years was RMB 724,373 and 657,236 respectively, and the former was ¥67,137 higher than the latter. Compared with MDI group, the expected life years of CSII group was higher (17.27 years vs. 16.86 years), and the QALYs was higher (11.61 years vs. 11.20 years), with a difference of 0.42 years. As shown in the **Table 4**, although the treatment cost of CSII group is higher than that of MDI group, the treatment cost of related complications is significantly lower than that of MDI group, especially for renal complications and severe hypoglycemia events. The ICER of the CSII group is ¥161,815/QALY, that is, the average cost of each additional QALY is ¥161,815, which is less than 1.5 times the per capita GDP of Qingdao in 2019 (RMB 186,423). Therefore, compared with MDI therapy, CSII is more cost-effective. **Table 5** shows the base analysis results of CSII and MDI groups.

**Table 5:** The base analysis results of CSII and MDI groups.

Items	CSII	MDI	Difference
Life year (Year)	17.27	16.86	0.41
QALYs	11.61	11.20	0.42
Direct medical costs	724,373	657,236	67,137
Treatment Cost	312,794	124,656	188,138
Disease Management	12,532	12,379	153
CVD complications	90,540	89,644	896
Renal complications	150,044	250,700	-100,656
Ulcer/Amputation/Neuropathy	117,349	125,139	-7,790
Eye complications	11,002	14,312	-3,310
Non Severe Hypoglycemia	0	0	0
Severe Hypoglycemia	22,271	31,445	-9,174
Adverse Events	7,842	8,962	-1.120
(ICER)	¥161,815 (Cost-effective)		

#### Scenario Analysis: Different in Time Horizon

As shown in **Table 6**, under the scenarios of 60, 50, 40, and 30 years of time horizon simulation, ICER is lower than 3 times of Qingdao’s per capita GDP in 2019. Under the scenarios of 60 and 50 years of time horizon, ICER is lower than 1.5 times of Qingdao’s per capita GDP in 2019.

**Table 6:** Scenario analysis: Different in Time Horizon.

	QALYs			Direct medical costs			ICER (RMB)
	CSII	MDI	Difference	CSII	MDI	Difference	
Pump price is¥60,000, the upgrade price will remain unchanged. time horizon 60 Year	11.612	11.197	0.415	¥724,373	¥657,236	¥67,137	161,815 (Cost-effective)
Pump price is¥60,000, the upgrade price will remain unchanged. time horizon 50 Year	11.38	11.02	0.36	¥703,298	¥639,008	¥64,290	178,238 (Cost-effective)
Pump price is¥60,000, the upgrade price will remain unchanged. time horizon 40 Year	10.88	10.59	0.29	¥661,107	¥599,058	¥62,049	212,494 (Cost-effective)
Pump price is¥60,000, the upgrade price will remain unchanged. time horizon 30 Year	9.94	9.72	0.22	¥587,014	¥525,094	¥61,920	287,864 (Cost-effective)

2019 Per capita in China GDP For 70,892, 1.5 Times per person GDP For 106,338, 3 Times per person GDP For 212,676. 2019 Per capita Qingdao GDP For 124, 282, 1.5 Times per person GDP For 186,423, 3 Times per person GDP For 372,846.

#### Scenario Analysis: Different in Discount Rate in Each Updating Cycle

As shown in **Table 7**, compared with the MDI group (60 years of time horizon), pump price is ¥60,000, and the ICER of CSII is lower than 1.5 times of Qingdao’s per capita GDP in 2019 under the scenario of each upgrading price discount from constant to 20% with each average 7.7 years of updating cycle. CSII group is dominant under the scenario of each upgrading price discount from 25% to 30%.

**Table 7:** Different in discount rate in each updating cycle.

Item	QALYs					Direct medical costs				ICER(RMB)
	CSII	MDI		Difference		CSII	MDI	Difference		
Pump price is¥60,000, the upgrade price will remain unchanged, with 60 years of time horizon	11.612		11.197		0.415	¥724,373	¥657,236		¥67,137	161,815 (Cost-effective)
Pump price is¥60,000 and the price of each upgrade cycle is reduced by 5% with 60 years of time horizon	11.612		11.197		0.415	¥703,431	¥657,236	¥46,195		111,340 (Cost-effective)
Pump price is¥60,000 and the price of each upgrade cycle is reduced by 10% with 60 years of time horizon	11.612		11.197		0.415	¥686,226	¥657,236	¥28,989		69,870 (Cost-effective)
Pump price is¥60,000 and the price of each upgrade cycle is reduced by 15% with 60 years of time horizon	11.612		11.197		0.415	¥672,096	¥657,236	¥14,860		35,815 (Cost-effective)
Pump price is¥60,000 and the price of each upgrade cycle is reduced by 20% with 60 years of time horizon	11.612		11.197		0.415	¥660,522	¥657,236	¥3,285		7,918 (Cost-effective)
Pump price is¥60,000 and the price of each upgrade cycle is reduced by 25% with 60 years of time horizon	11.612		11.197		0.415	¥651,032	¥657,236	¥-6,204		-14,953 (Cost-effective)
Pump price is¥60,000 and the price of each upgrade cycle is reduced by 30% with 60 years of time horizon	11.612		11.197		0.415	¥643,264	¥657,236	¥-13,973		-33,701 (Cost-effective)

2019 Per capita Qingdao GDP For 124, 282, 1.5 Times per person GDP For 186,423, 3 Times per person GDP For 372,846. Exchange Rate$1 =¥6.95.

#### Sensitivity Analysis: Results Based on Qingdao Per Capita GDP in 2019

**Figure 3:**
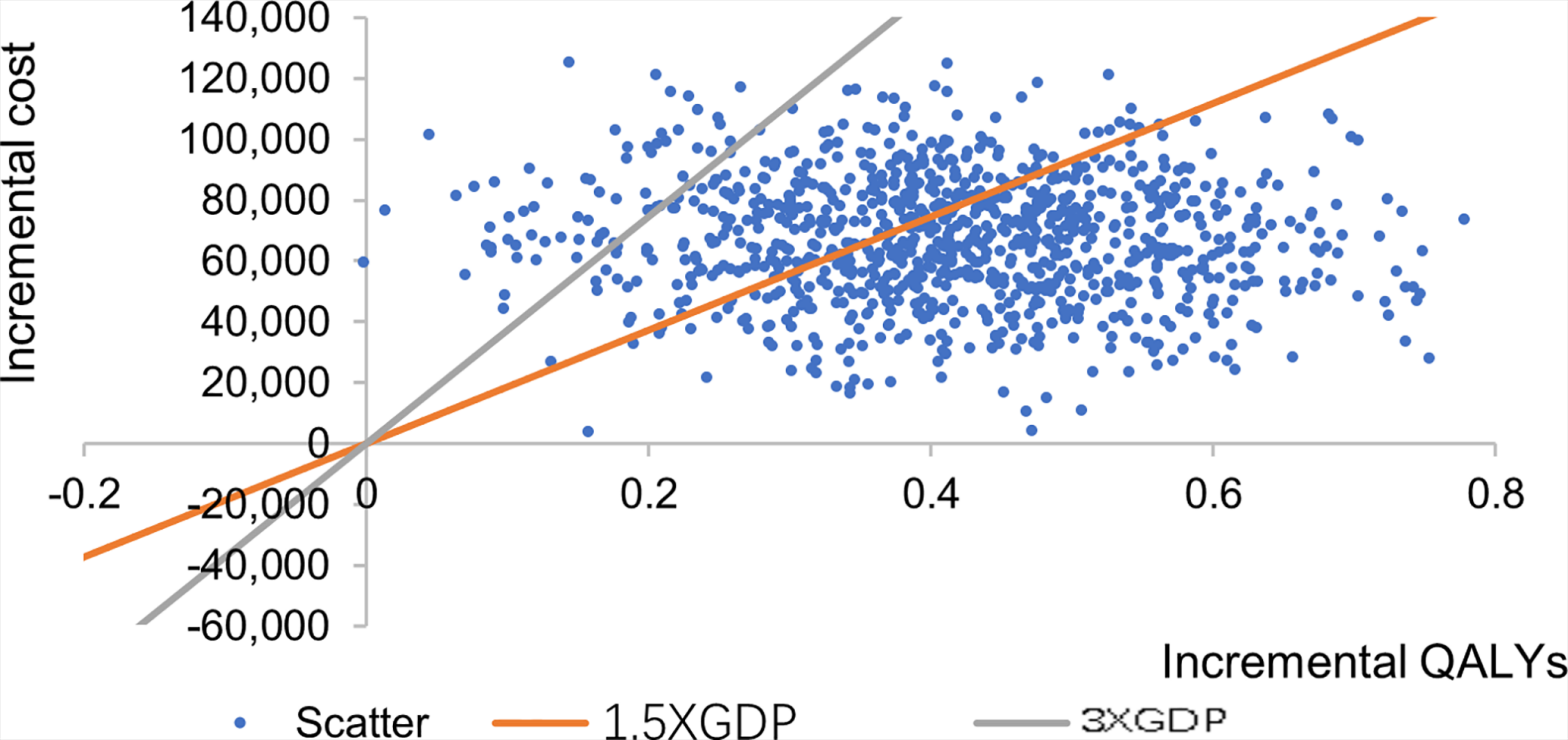
Incremental cost effect scatter diagram.

**Figure 4:**
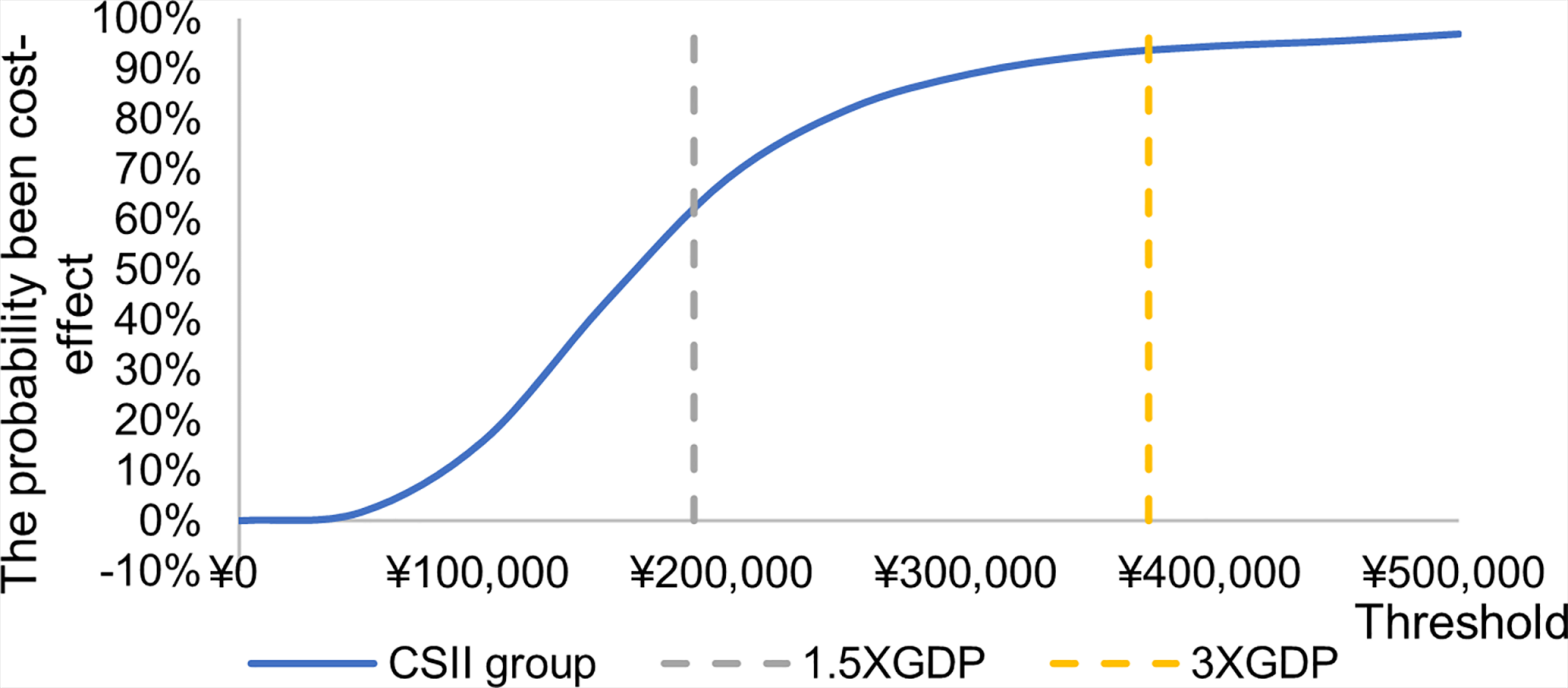
Cost effect acceptability curve.

The incremental cost effect scatter diagram (**Figure 3**) shows that when the GDP per capita of Qingdao is 1.5 times and 3 times as the threshold value, most of the scatter points fall below the threshold line, indicating that the probability of cost-effective in CSII group is higher than MDI group. The cost-effective acceptability curve (**Figure 4**) shows that under the 1.5 times and 3 times per capita GDP thresholds of Qingdao in 2019, the probability of CSII group been cost-effect is 62.8% and 93.7% respectively. This shows that the results of the basic analysis are robust.

#### Years of Diabetic Complication Onset

**Figure 5:**
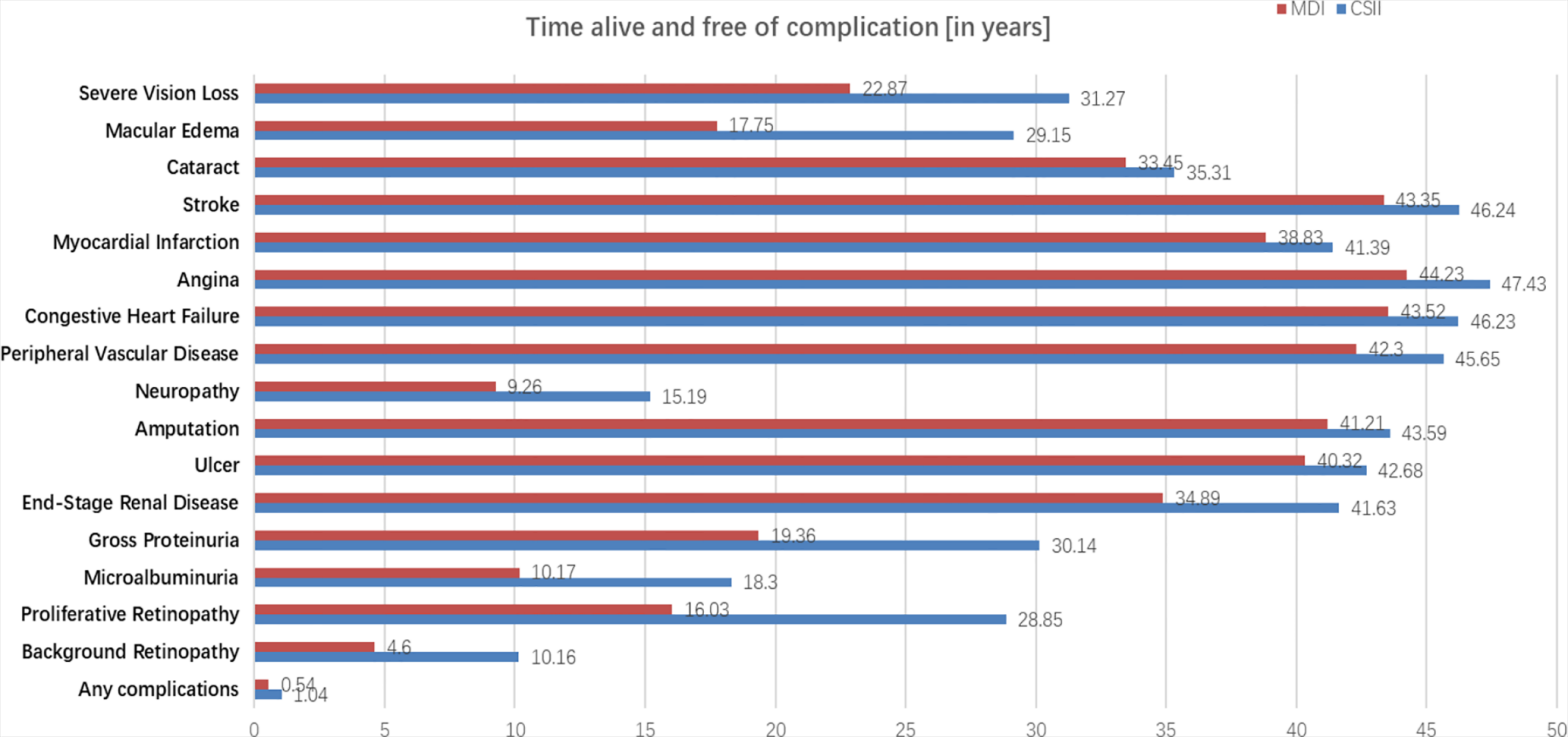
Time alive and free of complication (in years).

Compared with MDI group, CSII group can significantly delay the onset time of severe vision loss, macular edema, cataract, stroke, myocardial infarction, angina, congestive heart failure and other complications (**Figure 5**).

## Discussion

Results from studies investigating children and adolescent patients with T1DM suggested a better blood glucose control of CSII therapy compared with MDI therapy, as measured by a decrease in HbA1c at 1 year of treatment. In fact, at the end of the 1st year in the CSII group there was a significant decrease in HbA1c with 47.14% of the patients <7.5%, probably due to better enthusiasm of the patients in the 1st year. Although mean HbA1c levels were lower in the CSII group during follow up, the mean HbA1C level by the end of the 3rd year (8.68%) was found higher than the values at the end of the second (8.1%) and first (7.79%) years. The reasons of HbA1c level gradually increasing year by year are as follows: a) The average age of children with pumps at first diagnosis is 11 Age, gradually entering puberty, drastic changes in hormones can antagonize the glucose lowering effect of insulin. b) Most children with pumps are newly diagnosed and have 0.5–1 years of honeymoon period while the function of islets still remain partly, so the gap between in HbA1c control in two groups for the 1st year was less than other years. c) Patients behavior compliance to diseases management and treatment are gradually loosed. d) Children’s growth and development need looses the diet and behavior restrictions. This is consistent with findings from other studies (13–15) that showed CSII produces better HbA1c control at all times compared to MDI. In a meta-analysis (16) comparing CSII vs. MDI demonstrated that HbA1c has a greater reduction in patients treated with CSII in all trials. In one study (17) over a 5 year follow up period, HbA1c levels, despite improving in the first 1 year, tended to increase over the subsequent 4-year period in CSII patients keeping with our findings. Furthermore, a recent comparative study by Karges et al. (18) show that the use of CSII has better glycemic control during the most recent year of therapy. Similar results (19–22) have also been reported in other randomized controlled clinical trials, comparing the results of CSII and MDI in the treatment of T1DM, in which the HbA1C in CSII group was improved.

An analysis of mean daily insulin dose in both treatment groups showed significantly lower daily insulin requirements in CSII group after 1 and 4 years of treatment. Insulin delivery by CSII is closer to physiological insulin secretion, so it requires less insulin to achieve blood glucose control than MDI. This observation was reported in many other studies (23–26). As regards lipid profile, there was no statistically significant difference between CSII pump therapy and MDI injection regimens. In line with the results of our study, Ana et al. (27) reported that CSII can optimize metabolic control, reduce daily insulin demand, and achieve higher satisfaction without changing blood lipid level and increasing the frequency of adverse events. Similarly, in a study on Turkey adolescents, Ayhan et al. (8) demonstrated that triglyceride, total cholesterol, HDL- and LDL-cholesterol of CSII pump therapy were compared with MDI injection regimens, there was no statistically significant difference. Although there have been several studies reported lipid profiles were similar in the MDI and CSII groups, it should be noted that a short monitoring period has been a limitation in most of these studies.

As regards hypoglycemia and DKA episodes, there was a significant reduction in them in the CSII group compared to MDI throughout the study period. This may be due to the fact that insulin delivery from the insulin pump is more physiological, and the availability of multiple base rates based on activity, sleep, and diet patterns reduces the frequency of severe hypoglycemic events (24). Moreover, the observation on reduction in severe hypoglycemic episodes was consistent with many earlier studies (28–30). The reduction of DKA episodes depended on specific diabetes education training, flexibility of insulin application, better glycemic control. Conversely, some research (7, 14, 31) reported an increase in DKA episodes in the insulin pump therapy.

This study conducted the first cost-effectiveness analysis of CSII and MDI using health economics model and CORE mode, based on a cohort of T1DM pediatric patients treated in a public healthcare institution of China. Why we need the local Health economic data: a) Currently only Qingdao has long term reimbursement policy for insulin pump and Qingdao government needs to know the health economic benefits of this project. b) Health economic data generation in Qingdao can provide strong evidence to support reimbursement negotiations in other provinces of China. c) Evidence of foreign health economics cannot be directly applied to China and the actual clinical effectiveness of pump therapy will be different in different national conditions medical system and national environment. d) Real-world data are more meaningful. It is very important to study the evidence of safety, efficacy and cost-effectiveness of CSII in order to better understand its role and benefits in the management of type 1 diabetes. Through model, calculate the economic data such as QALY, ICER, and long-term treatment costs, and evaluate the health economic benefits of the Qingdao project.

Our study shows that the usage of CSII is an more effective therapy, although it has a higher overall cost of treating children with T1DM, and confirms similar results reported in the literature on improving metabolic control, reducing the incidence of complications and improving quality of life (32–35). Under the scenarios of 60 years of time horizon simulation, the long-term direct medical cost of the CSII group is only 67,137 yuan higher than that of the MDI group, because CSII treatment significantly reduces the treatment cost of diabetes-related complications, especially renal complications and severe hypoglycemia events. Meanwhile, this model reflects the delayed onset of diabetes-related complications, which affects clinical and economic outcomes. Compared with MDI, CSII treatment in children with T1DM has an increase of 0.41 Life Years and 0.42 QALYs, respectively. CSII can improve quality of life and increase health output. CSII is associated with an ICER of ¥ 161,815/QALY gained compared with MDI, which is lower than 1.5 times of Qingdao’s per capita GDP in 2019. In addition, the key influential factors of health economics model were tested by multiple sensitivity analysis. Under the scenarios of 30, 40, 50, and 60 years of time horizon simulation, ICER is lower than 3 times of Qingdao’s per capita GDP in 2019,however,under the scenarios of 30 and 40 years, ICER is more than 1.5 times of per capita GDP. The results of this study are consistent with other studies that evaluated the efficacy and ICER of insulin pump therapy in developed countries (34, 36, 37). The findings show that compared with MDI, long-term or even life-long CSII treatment has cost-effective advantages. And the sensitivity analysis also shows that the result of basic analysis is robust (62.8%). Although these figures do not represent the CSII-treated T1D pediatric population in the whole country, it does suggest that there is likely to be a considerable number of patients in China for whom switching to CSII would be clinically beneficial and cost-effective.

The main limitation of this study is the retrospective cohort study, lacking of randomized and crossover design between the CSII and MDI group. Although the retrospective analysis has some missing data, it still truly reflects the difference between the two groups. In addition, hypoglycemia was measured by self-monitored blood glucose rather than continuous glucose monitoring, so nighttime and asymptomatic hypoglycemia may not be fully determined, which may cause deviations in results. A potential limitation of health economic analysis is that due to the lack of research data on the treatment cost of T1DM related complications in China, we mainly refer to the published direct medical cost report of T2DM related complications in China. Finally, this is a single-center study and the sample size is small.

## Conclusion

CSII offers advantages over MDI treatment, such as better control of glucose levels reflected by HbA1c. Other advantages were reduced hypoglycemia and DKA events. In conclusion, this study is of importance because it provides the results of using CSII pump therapy in children and adolescents for the first time in China. Especially in developing country, the actual clinical effectiveness and cost-effectiveness of pump therapy can prompt governments to support reimbursement policy for insulin pump.

## Data Availability Statement

The datasets generated for this study are available on request to the corresponding author.

## Ethics Statement

The studies involving human participants were reviewed and approved by Qing Dao Women and Children’s Hospital Ethical committee (QFELL-KY-2019-61). Written informed consent to participate in this study was provided by the participants’ legal guardian/next of kin.

## Author Contributions

SH and HY conceived and designed the study, contributed to the analysis, interpreted the results, and wrote the manuscript. All authors contributed to the article and approved the submitted version.

## Funding

This work was supported by the National Natural Science Foundation of China (81170762).

## Conflict of Interest

Author WL was employed by Medtronic (Shanghai) Management Co, Ltd.

The remaining authors declare that the research was conducted in the absence of any commercial or financial relationships that could be construed as a potential conflict of interest.
